# Macroscale pseudo-spheroids fabricated using methacrylated collagen-coated cells

**DOI:** 10.7150/thno.92193

**Published:** 2024-01-01

**Authors:** SooJung Chae, Hyeongjin Lee, Dongryeol Ryu, GeunHyung Kim

**Affiliations:** 1 Department of Precision Medicine, Sungkyunkwan University School of Medicine (SKKU-SOM) Suwon 16419, Republic of Korea.; 2 Department of Biotechnology and Bioinformatics, Korea University, Sejong, Republic of Korea.; 3 Biomedical Science and Engineering, Gwangju Institute of Science and Technology, Gwangju, Republic of Korea.; 4 Institute of Quantum Biophysics, Department of Biophysics, Sungkyunkwan University, Suwon, Gyeonggi-do 16419, Republic of Korea.; 5 Biomedical Institute for Convergence at SKKU (BICS), Sungkyunkwan University, Suwon 16419, Republic of Korea.

## Abstract

**Rationale:** Cell spheroids have shown great promise as tools for creating effective three-dimensional (3D) tissue models, facilitating tissue reconstruction and organoid development, due to their high cell density and efficient cellular interactions. However, a significant challenge persists in creating large-scale tissue structures with a 3D geometrical architecture using spheroids, due to the continual condensation and reorganization of cells and their environments.

**Methods:** The spherical cell aggregates (pseudo-cell spheroids) or macroscale cell aggregates were obtained by coating each adipose-derived stem cell (hASC) with methacrylated collagen (Col-Ma). Subsequently, the coated cells were printed into an alginate supporting bath and photocrosslinked through exposure to UV light. To assess the effectiveness of this procedure on regenerative potential, the generated cell aggregates were compared with conventional cell spheroids and bioprinted cell constructs using immunofluorescent staining and quantification of myogenic-related gene expressions. Moreover, the bioconstructs were implanted into a mouse model with volumetric muscle loss to further elucidate their regenerative and functional recovery properties.

**Results:** The use of Col-Ma as a cell-coating material enables the rapid and physical aggregation of cells within several hours, regardless of the cell type. Furthermore, Col-Ma-coated cell aggregates can provide relatively lower hypoxic conditions than cell spheroids fabricated using the hanging drop method owing to the thin porous Col-Ma layer coated on the cells. In addition, the resulting structures maintain their geometrical architecture following cell fusion and possess the potential for efficient scale-up and 3D complex shape formation, making them more suitable for clinical applications than conventional cell spheroids. Finally, the feasibility of the Col-Ma-coated cylindrical human adipose-derived stem cells aggregates was assessed through implantation in a mouse volumetric muscle loss model, showing a significantly higher regenerative ability of muscle tissue than the normally bioprinted cell construct.

**Conclusion:** Our newly proposed method has meaningful potential for various tissue engineering applications, supported by the improved cellular activities and efficient muscle regeneration observed in both *in vitro* and *in vivo* studies, and organ-chip models.

## Introduction

Tissue engineering is a rapidly progressing field that focuses on creating functional tissues and organs using biomaterials, cells, and biophysical and biochemical factors 1. Cell spheroids have emerged as promising tools for developing effective three-dimensional (3D) tissue models, tissue reconstruction, and organoids owing to their high cell density and efficient cellular interactions 2, 3. This is particularly important when creating constructs that mimic high-cell-density tissues, such as cardiac tissue, or developmental processes, such as N-cadherin cell-cell interactions during mesenchymal condensation 4, 5.

Cell spheroids are typically formed using various cell clustering processes, in which cells adhere to each other instead of to a substrate. Popular methods for creating cell spheroids include the hanging drop and micro-well techniques, while other techniques utilize micro-rotational flow or the magneto-Archimedes effect to produce uniformly sized tissue spheroids 6-9. With conventional methods, spheroids are formed from concentrated cells through gravity and cell aggregation after several days of culture 10. However, the fabrication of cell spheroids with consistent sizes and shapes has been challenging because spheroid formation can be directly influenced by factors such as cell type, seeding density, culture conditions, and the spheroid formation method 10.

Spheroid formation involves three key steps: initial loose cellular aggregation driven by integrin-ECM interactions, subsequent accumulation of cadherin, and compact spheroid formation through cadherin-cadherin interactions 11-13. Briefly, essential cell adhesion molecules, such as cadherins and integrins, play pivotal roles in this process. In the initial stage, single cells bind to the ECM through membrane integrins, inducing weak aggregation. E-cadherin, a principal glycoprotein in the Ca^2+^-dependent system, can facilitate homotypic cell-cell adhesion and trigger robust spheroid compaction upon cell accumulation. For efficient spheroid formation, the aspiration of cells within a hydrogel has been commonly employed. For example, Jeon et al. have developed a "3D bio-dot printing" system in which cells are deposited within a sacrificial hydrogel, generating the aggregation of cells to form spheroids 14.

Recent advances in biomanufacturing methods, including bioprinting, have enabled the fabrication of complex 3D structures with high accuracy and reproducibility. The development of functional bioinks combined with cell spheroids has great potential 15. Biofabrication techniques, such as controlled seeding of spheroids into macroporous scaffolds, aspiration-assisted bioprinting, and extrusion-based bioprinting, have been proposed to control spheroid deposition 4, 16, 17. These methods provide a means to create 3D artificial tissues with spheroids acting as building blocks in tissue formation. Generally, successful regeneration of 3D tissue constructs consisting of cell spheroids relies heavily on their fusion and maturation into functional macroscale tissue constructs 4. The fusion of multiple spheroids typically occurs within 1-3 days, driven by differences in the interfacial surface tension between the surrounding culture media and cells 18. This fusion process can be influenced by several factors, including extracellular matrix secretion, actin dynamics, and the formation of a condensation boundary over time 19, 20. Additionally, for successful spheroid fusion, the spheroid contact should occur before forming a condensation boundary, typically within seven days for human mesenchymal stromal cells 21. Failure to achieve spheroid contact before setting a condensation boundary can restrict the fusion of interspheroids and result in loosely connected spheroid aggregation 22. Despite following these strategies, producing macroscale tissue structures that maintain a 3D geometrical architecture following spheroid fusion remains a significant challenge because of the constant condensation and reorganization of cells and their environments.

In this study, we present a new method for the fabrication of spherical cell aggregates (pseudo-cell spheroids) or macroscale fibrous cell aggregates using human adipose-derived stem cells (hASCs) and methacrylated collagen (Col-Ma) solution. To achieve the macroscale cell aggregates, each cell was coated with low weight faction (0.03 wt%) of Col-Ma solution, and the Col-Ma-coated cells were laden in an alginate supporting bath (6 wt%). The printed cells were then exposed to ultraviolet (UV) light and were in close contact with the hydrostatic force of the alginate solution and proper diffusion of media to form cell aggregates.

To demonstrate the feasibility of this process, we printed Col-Ma-coated hASCs onto an alginate solution to fabricate cylindrical cell aggregates similar to the shape of a microscale baguette (diameter = 603 ± 60 µm and length = 10 ± 1 mm). The fabricated structure was implanted in a mouse volumetric muscle loss (VML) model, and we observed a significantly higher regenerative ability of the muscle tissue compared with that of the cell struts, which were fabricated using a submerged bioprinting process with a bioink consisting of cell density (hASC, 1 × 10^7^ cells/mL) and Col-Ma (5 wt%).

This method offers several advantages over conventional spheroid fabrication methods and techniques using spheroids to obtain macroscale tissue structures. Using Col-Ma as a cell-coating material allows for significantly rapid and firm cell aggregation within several hours, independent on cell types. Furthermore, unlike the previous two-step processes of spheroid fabrication and the fusion of multiple spheroids to fabricate 3D macroscale tissue constructs, this method can fabricate a macroscale structure in a one-time process using complex 3D shapes that can be obtained using a general submerging printing process.

Based on these results, our method for fabricating cell aggregates using Col-Ma-coated cells and submerged printing can potentially advance tissue engineering. In particular, this method has the potential for scaling up and automation, making it suitable for clinical applications.

## Materials and Methods

### Synthesis of Col-Ma

Col-Ma was synthesized as previously described 23. Briefly, porcine-derived type I collagen (MS bio, South Korea) was dissolved in 0.5 M acetic acid, and 10 M sodium hydroxide was gently added to adjust the pH to 8-9. Subsequently, 0.2 mL of methacrylic anhydride per 100 mg of collagen was added to the solution and stirred for three days. To precipitate methacrylated collagen, 20 mL of acetone was added to 30 mL of the collagen solution. After centrifugation, the collected Col-Ma was dialyzed in deionized water using dialysis tubing (molecular cut-off of 3.5 kDa; Spectrum Labs, Inc., USA) at 4 °C for three days to remove the remaining chemical molecules and was lyophilized. Freeze-dried Col-Ma was stored at -80 °C before use.

To determine the degree of methacrylation of Col-Ma, ninhydrin assay was conducted using previously outlined protocol 24. Briefly, ninhydrin solution (5 w/v%) was mixed with ethozyethanol and 200 mM citric acid in 0.16 % (w/v) tin II chloride to prepare the reaction solution. Then, the 2.5 mg of the prepared Col-Ma was reacted with 1 mL of the prepared solution for 15 min at 100 ^o^C. Subsequently after the treatment, 250 μL of isopropanol (50 v/v% diluted with tripled distilled water) was added to terminate the reaction. Finally, the solution was inserted into 96-well plate and a microplate reader (EL800; BioTek, USA) was used to measure the absorbance at 570 nm.

### Cell culture and fabrication of hASC-spheroids

hASCs were purchased from Lonza (Switzerland) and cultured in low-glucose Dulbecco's modified Eagle's medium (DMEM; Welgene, South Korea) supplemented with 10% fetal bovine serum (Biowest, France) and 1% penicillin/streptomycin (Gibco, USA). The medium was changed every two days. To compare the fabricated cell aggregates (experimental group), conventional hASCs spheroids (control group) were fabricated *via* the hanging drop method 6. Briefly, 1 × 10^5^ hASCs suspended in 20 µL of medium were dropped onto the inside of the lid of a cell culture plate (SPL, South Korea). The lid of the plate was then gently inverted over a cell culture dish filled with phosphate-buffered saline (PBS) and cultured for three days to generate cell spheroids.

### Cell-coating process using Col-Ma and the fabrication of cell aggregates

To fabricate cell aggregates, the cells were coated with Col-Ma using a modified version of a previously described method 25. The Col-Ma solution was prepared by dissolving lyophilized Col-Ma in 5 mM acetic acid. Subsequently, 1 × 10^6^ cells were coated by mixing 400 µL of Col-Ma solution with 600 µL of cell-suspended media. The coating process took place in a rocker shaker (JeioTeck, South Korea) at 37 °C for 1 h. After the coating process, the solution was centrifuged, and the supernatant was removed. The cells were then suspended in 10 µL of 10×DMEM solution containing 5 µg/µL lithium phenyl (2,4,6-trimethylbenzoyl) phosphinate (LAP; Sigma-Aldrich, USA). To prepare the alginate solution used in the supportive bath printing solution, sodium alginate (LF10/60; FMC Biopolymer, Norway) was dissolved in triple-distilled water and mixed with 10×DMEM at 9:1 to obtain a 6 wt% alginate solution. The alginate solution was then poured into a 35 mm Petri dish (SPL, South Korea), and the Col-Ma-coated cells mixed with media were injected into the alginate bath. To crosslink the cell aggregates, the cells were exposed to UV light (LIM Technology, South Korea) (570 mJ/cm^2^) and cultured in an alginate bath for three days, followed by alginate bath removal.

### Fabrication of the bioprinted cell struts

To compare the cell aggregates (EXP-2) with the conventional cell-printed structure (EXP-1), hASC-laden Col-Ma bioink was printed using a printing system (DTR2-2210T-SG; DASA Robot, South Korea) supplemented with a dispenser (AD-3000C; Ugin-tech, South Korea). The lyophilized Col-Ma was dissolved in 0.1 M acetic acid and neutralized with 10 × DMEM at 1:1 to reach a final concentration of 5 wt%. Then, 1 × 10^7^ cells/mL were mixed with the bioink and extruded into a supportive alginate bath through a nozzle (inner diameter: 500 µm). The alginate bath was then removed.

### In vitro test

To evaluate cell viability, the cells on the samples were treated with 0.15 mM calcein AM (Invitrogen, USA) and 2 mM ethidium homodimer-1 (Invitrogen) at 37 °C for 60 min. To observe the cytoskeleton of the cultured cells laden in the fabricated structures, cell nuclei and F-actin were stained. Before staining, cultured cells were fixed with 3.7% formaldehyde (Sigma-Aldrich) for 1 h to facilitate fixation. Subsequently, 0.1% Triton X-100 (Sigma-Aldrich) was used to permeabilize the cell constructs, and the samples were stained for 1 h with diamidino-2-phenylindole (DAPI; 1:100 dilution in PBS; Invitrogen) for cell nuclei and Alexa Fluor 488 phalloidin (1:100 dilution in PBS; Invitrogen) for F-actin. To confirm the regularity of Col-Mya-coated cells, the cells were fixed and stained with DAPI and Picro-Sirius red (Sigma-Aldrich) for 1 h at 37 °C and washed twice with triple-distilled water. The stained cells were visualized under a confocal microscope (LSM 700; Zeiss, Germany), and ImageJ software was used to calculate cell viability, cell number, and diameter of the fabricated cell constructs.

To stain the spheroids and cell aggregates with hypoxia-inducible factor-1α (HIF-1α) immunofluorescent staining, the samples were treated with 3.7% formaldehyde for 1 h and 2% Triton X-100 for 1 h. Then, the cell constructs were immersed in bovine serum albumin (Sigma-Aldrich) for 2 h at 37 °C to block the non-specific antibody binding. Subsequently, the cell constructs were incubated with an anti-HIF-1α primary antibody (5 μg/mL, Abcam, USA) overnight at 4 °C and stained with DAPI and Alexa Fluor 488-conjugated secondary antibodies (1:50 in PBS, Invitrogen). The stained cells were visualized using a confocal microscope.

To observe the morphology of the cells and generated structures, the samples were fixed with 3.7% formaldehyde and washed with triple-distilled water. The samples were evaluated using scanning electron microscopy (SEM) (SNE-3000 M; SEC Inc., South Korea).

### Real-time polymerase chain reaction (RT-PCR)

Gene expression levels in cell aggregates and spheroids were compared using quantitative real-time polymerase chain reaction (RT-PCR). RNA was isolated using the Easy-Blue reagent (Intron Biotechnology, South Korea). The purity and quantity of the RNA were assessed using a spectrophotometer (FLX800T; Biotek, USA). ReverTra Ace qPCR RT Master Mix (Toyobo Co., Ltd., Osaka, Japan) synthesized complementary DNA (cDNA) from RNase-free DNase-treated total RNA. Then, the reactions were performed using SYBR (Thunderbird® SYBER®; Toyobo Co., Ltd., Japan) and a Plus real-time PCR system (StepOnePlus; Applied Biosystems, USA). Glyceraldehyde 3-phosphate dehydrogenase (GAPDH) was used as a housekeeping gene to normalize expression levels. Specific gene expression are as follows: human GAPDH (forward: 5′- GGA TTT GGT CGT ATT GGG -3′, reverse: 5′- GGA AGA TGG TGA TGG GAT T -3′), human N-cadherin (forward: 5′- ACA GTG GCC ACC TAC AAA GG -3′, reverse: 5′- CCG AGA TGG GGT TGA TAA TG -3′), human integrin β1 (forward:5′- GAA GGG TTG CCC TCC AGA -3′, reverse: 5′- GCT TGA GCT TCT CTG CTG TT -3′), human connexin 43 (forward: 5′- GCC TGA ACT TGC CTT TTC AT -3′, reverse: 5′- CTC CAG TCA CCC ATG TTG C -3′), human fibronectin (forward: 5′- CGG TGG CTG TCA GTC AAA G -3′, reverse: 5′- AAA CCT CGG CTT CCT CCA TAA -3′), human HIF 1α (forward: 5′- CTC AAA GTX GGA CAG CCT CA -3′, reverse: 5′- CCC TGC AGT AGG TTT CTG CT -3′), human OCT4 (forward: 5′- GCA GCG ACT ATG CAC AAC GA-3′, reverse: CCA GAG TGG TGA CGG AGA CA-3′), and human SOX2 (forward: 5′- CAT CAC CCA CAG CAA ATG ACA-3′, reverse: GCT CCT ACC GTA CCA CTA GAA CTT-3′), were purchased by the bionics (South Korea).

### *In vivo* procedure

Tibialis anterior (TA) muscle defects were induced in male C57BL/6 mice (10 weeks old, DooYeol Biotech, Inc., Seoul, Korea). All animal procedures were approved by the Institutional Animal Care and Research Advisory Committee of the Sungkyunkwan University School of Medicine Laboratory Animal Research Center and complied with the regulations of the Institutional Ethics Committee (SKKUIACUC2021-08-11-2).

Before surgery, mice were randomly divided into four groups (16 mice, n = 4/group). The mice were anesthetized using 3% v/v isoflurane, and the left and right hind legs were depilated using a sterile blade. The skin was then incised approximately 4 mm, and the extensor digitorum longus and extensor hallucis longus muscles were removed to separate the muscle from the fascia and prevent compensatory hypertrophy during muscle regeneration. Approximately 40% of the TA muscle was excised and weighed (supporting information, Table S1). To facilitate VML restoration, the TA muscle defect regions were transplanted with cell constructs. The transplanted area was then covered with sutured fascia and skin. Following the procedure, mice were administered a subcutaneous injection of 0.5 mg/kg buprenorphine for pain management and provided regular food and water. After a 4-week implantation period, euthanasia was performed by CO_2_ inhalation, followed by secondary cervical dislocation. Before transplantation, the cell constructs were cultured in a growth medium for one day to ensure cell stabilization 26, 27.

### Muscle functional evaluations

The skeletal muscle functionality of mice subjected to various treatments was evaluated by assessing hind limb grip strength and latency to fall. To measure hind limb grip strength, a grip strength meter (BIO-GS3, BIOSEB, FL, USA) was used four weeks after transplantation to determine the maximum force exerted 28. Briefly, the mice gripped a metal T-bar (BIO-GRIPBS Bar for mice) with their hind paws, and the maximum force was measured by applying parallel pulling until release 29. Furthermore, latency to fall was measured by recording the time elapsed after placing the mouse on a rod, with a maximum latency period of 300 seconds. Each experiment was repeated three times per mouse, with a 5-minute interval between repetitions. Four weeks after implantation, the TA muscles were excised from the mice and weighed.

### Histological and immunofluorescent staining

For histological staining, the harvested TA muscle samples were fixed in 10% neutral-buffered formalin for 24 h. Subsequently, the samples underwent dehydration, paraffin embedding, and sectioning into slices with a thickness of 5 µm. Muscle sections were stained with hematoxylin and eosin (H&E) and Masson's trichrome (MT). The resulting H&E- and MT-stained images were analyzed using ImageJ software to quantify myofiber diameter, myofiber area, and fibrotic area (n = 4 per sample, with four random fields assessed in each sample).

For immunofluorescence staining, the sectioned samples were subjected to overnight incubation at 4 °C with the following primary antibodies: anti-myosin heavy chain (MHC; MF20, 5 μg/mL, Developmental Studies Hybridoma Bank, Iowa City, IA), anti-human leukocyte antigen (HLA)-A (species reactivity: human, 1:100 dilution; Abcam, Cambridge, UK), anti-cluster of differentiation 31 (CD31; 1:200 dilution, Invitrogen, USA), anti-neurofilament (NF, 1:1000 dilutions; Abcam), anti-cholinergic receptor nicotinic beta 2 (CHRNB2; 1:100 dilution, Thermo-Fisher Scientific, USA), anti-F4/80 (1:100 dilution, Abcam), anti-I-A/I-E (MHCII; 1:100 dilution, Biolegend, San Diego, USA), and anti-CD206 (1:100 dilution, Biolegend). The samples were then washed with Dulbecco's phosphate-buffered saline (DPBS) and incubated with the following secondary antibodies: Alexa Fluor 488-conjugated (green; 1:50 in DPBS; Invitrogen) or Alexa Fluor 594-conjugated (red; 1:50 in DPBS; Invitrogen), and Alexa Fluor 488-conjugated anti-rabbit IgG (1:200 dilution, Invitrogen) for 1 h. Nuclei were stained with DAPI. Immunofluorescence images were captured using confocal microscopy. HLA-A-positive cells (%) were quantified by analyzing double immunofluorescence images for MHC/HLA-A (×400 magnification) in a blinded manner (n = 4 per sample, with four random fields assessed in each sample). The vascularization of the implanted constructs was evaluated by measuring the vessel area (%) using DAPI/CD-31 immunofluorescence images (×400 magnification) in a blinded manner (n = 4 per sample, with four random fields assessed in each sample). Neuromuscular junction (NMJ) formation was assessed using double immunofluorescence for NF/CHRNB2 by measuring the number of NMJs per field (%) (×400 magnification, n = 4 per sample, with four random fields assessed in each sample). To assess the immune response, immunofluorescence using F4/80 (a macrophage marker) was used to quantify F4/80 positive cells (%) (n = 4 per sample; four random fields were evaluated in each sample). All sample images were quantified using the ImageJ software. Results are presented as mean ± standard deviation.

### Statistical analysis

All experiments were repeated at least three times and are presented as the mean ± standard deviation. Statistically significant differences among the groups were determined using SPSS 18 software (SPSS, Inc., Chicago, IL, USA). Student's t-test was used to compare two groups. Additionally, to compare more than two groups, an analysis of variance (ANOVA) was performed, followed by Tukey's post hoc test. p-values of ^*^ *p* < 0.05, ^**^ *p* < 0.005, ^***^ *p* < 0.001, and ^****^ *p* < 0.0001 were considered statistically significant.

## Results and Discussion

### Cell spheroid formation using Col-Ma-coated cells

Cell spheroids are becoming increasingly popular in biomedical research because of their more physiologically relevant environment and improved cell-to-cell communication compared to traditional two-dimensional cell cultures. Spheroids have been fabricated using various methods, including seeding and culturing cells onto a non-adhesive surface, where they self-assemble into spherical clusters, hanging drops, agitation-based approaches, micropatterns, and 3D bioprinting 30. However, conventional spheroid fabrication methods have limitations such as time-consuming processes, non-uniform spheroid size, difficulties retrieving spheroids, and mechanical stress for extrusion 31.

**Figure 1:**
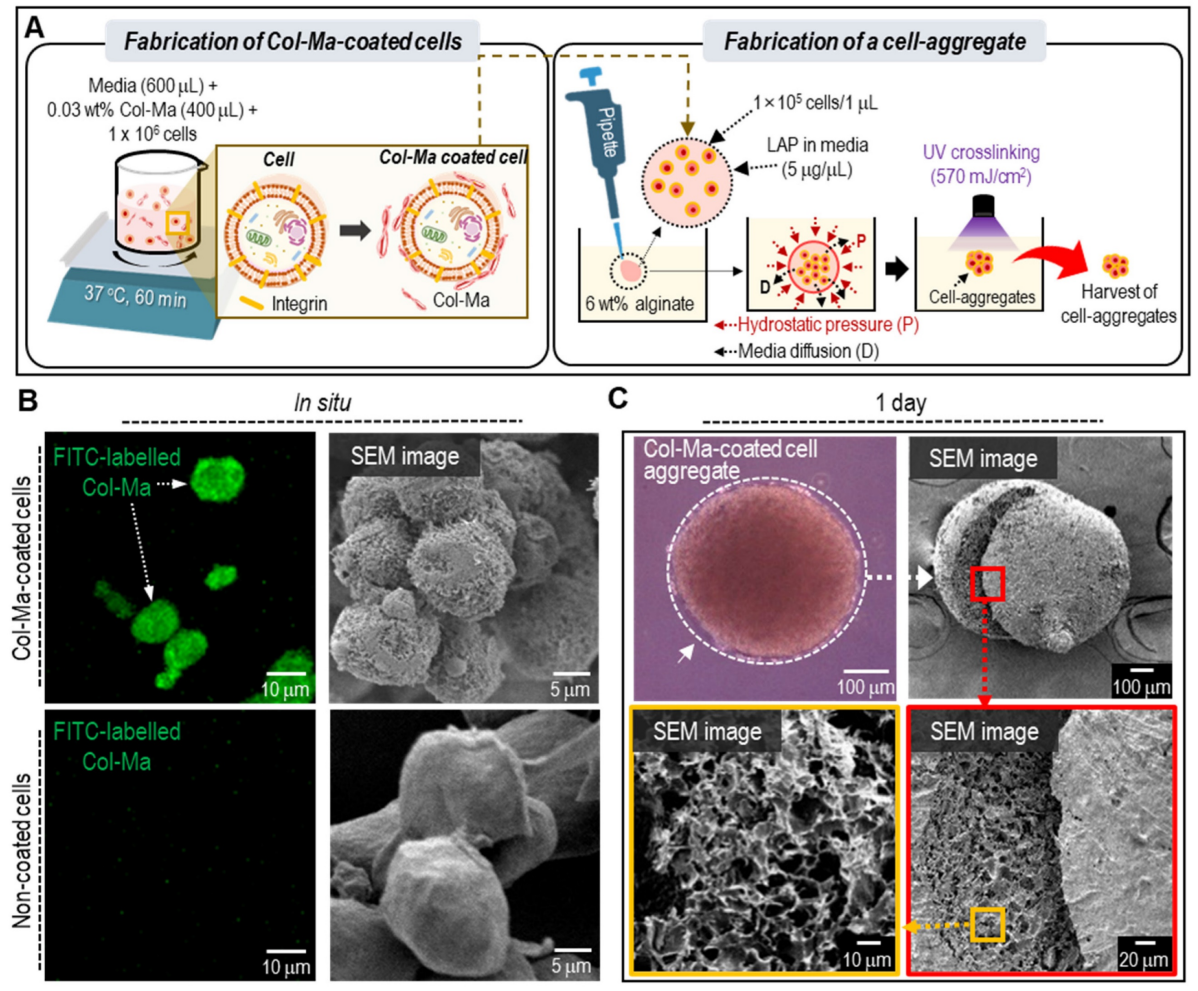
**(A)** A schematic describing the fabrication of the cell aggregates through cell-coating with Col-Ma and submerged printing in alginate solution. **(B)** Comparison of fluorescence and SEM images between Col-Ma-coated cells and normal cells. **(C)** Optical, surface and cross-sectional SEM images of a cell aggregate using the Col-Ma-coated cells.

In this study, we proposed a new method for cell spheroid fabrication using Col-Ma-coated cells. As shown in **Figure 1A**, cells were coated with a specific concentration (0.03 wt%) of Col-Ma and mixing time (60 min). The evaluation of the relative free amine group, as determined by the ninhydrin assay, indicated a significant decrease after the methacrylation process. This suggests that a large proportion of the free amine groups have undergone modification **(Figure S1A)**. Additionally, the rheological properties (complex viscosity, η*) and stress-strain curves, as illustrated in **Figure S1B-C**, indicate that the rheological and mechanical properties of Col-Ma have increased after the UV photocrosslinking process. Then the coated cells mixed with a specific medium volume were immersed in an alginate bath. Hydrostatic pressure and diffusion of the medium can induce the aggregation of cells. The aggregated cells were inflexibly photo-crosslinked with a thin-coated Col-Ma layer.

**Figure 1B** shows the fluorescence and SEM images of cells (hASCs) coated with and without fluorescein isothiocyanate (FITC)-labeled Col-Ma. In the images, the Col-Ma-coated cells showed a clear green color of fluorescein isothiocyanate, indicating that the cells were well-coated with Col-Ma. Furthermore, the SEM image showed a roughened surface of Col-Ma-coated cells, indicating that the Col-Ma layer covered the cells well, whereas uncoated cells showed very smooth cell surfaces. After Col-Ma coating, hydrostatic pressure and medium diffusion occurred in the alginate solution, and crosslinking with UV light resulted in spherical cell aggregates, which were easily retrieved from the alginate solution. Optical, surface, and cross-sectional SEM images of the resulting cell aggregates are presented in **Figure 1C**.

**Figure 2:**
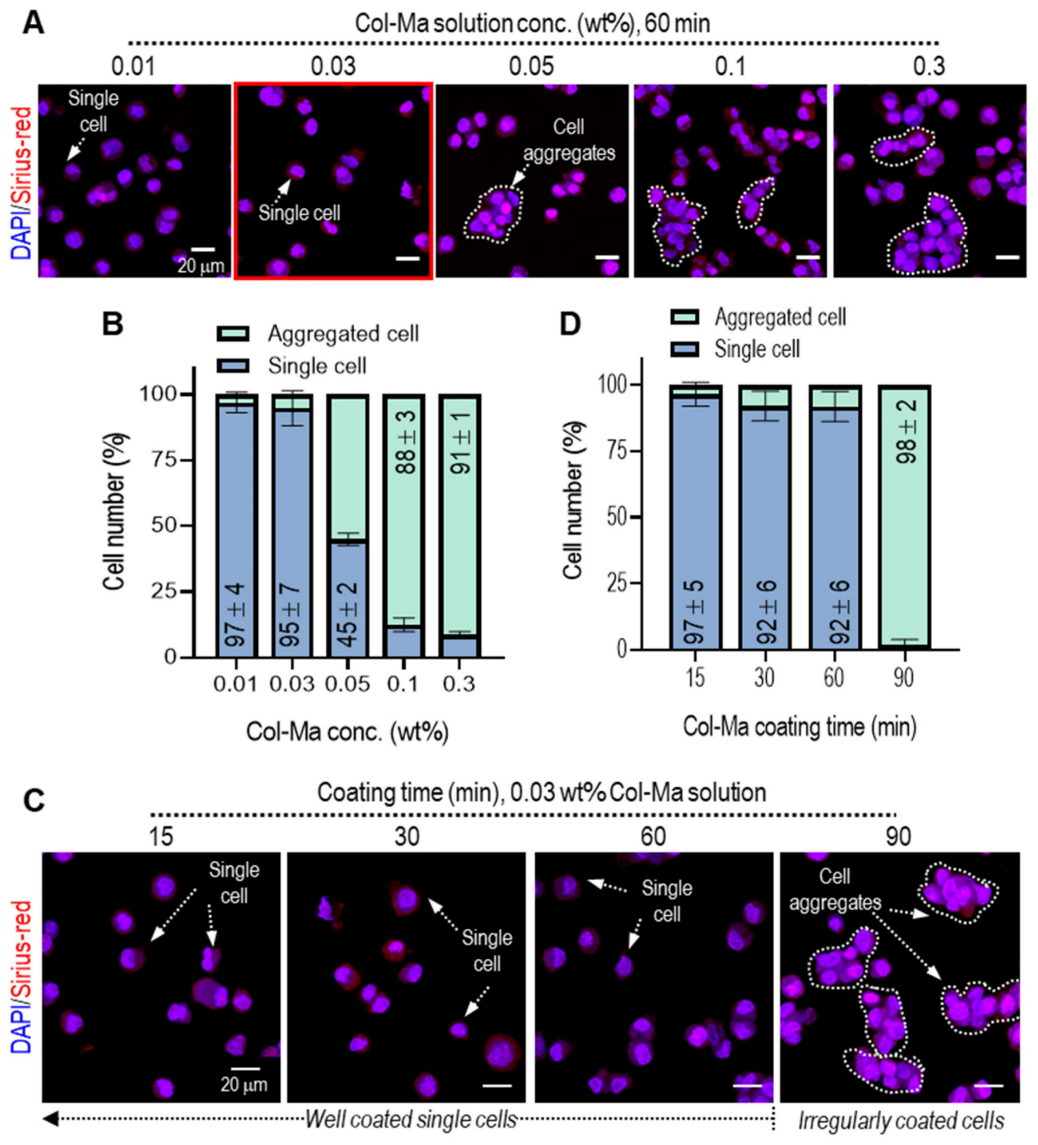
**(A)** Fluorescence images (DAPI: blue and Picro-Sirius red: red) of Col-Ma-coated cells for various Col-Ma solutions under a fixed mixing time (60 min) and **(B)** the ratio of irregularly grouped cells and single cells during the cell-coating process for various Col-Ma concentration. **(C)** Fluorescence images of Col-Ma-coated cells for various mixing times under a fixed Col-Ma concentration (0.03 wt%) and **(D)** the ratio of irregularly grouped cells and single cells for various coating times.

It is important to obtain single cells homogeneously coated with Col-Ma solution because irregularly grouped cells can prevent the stable spherical formation of pseudo-spheroids. For this reason, we determined the established Col-Ma coating conditions to obtain homogeneously coated single cells. To observe the effect of Col-Ma concentrations on the homogeneous coating of the cells (hASCs), various concentrations from 0.01 to 0.3 wt% and coating times from 15 to 90 min were used, as shown in **Figures 2A-D**. The coated cells were irregularly grouped with increasing Col-Ma concentrations and coating times (**Figures 2B, D**). However, in the coating condition of ~0.03 wt% of Col-Ma and ~ 60 min, homogeneous distributed and coated single cells were observed. From the results, we fixed the Col-Ma coating condition of the cells at 0.03 wt% of Col-Ma and a coating time of 60 min.

**Figure 3:**
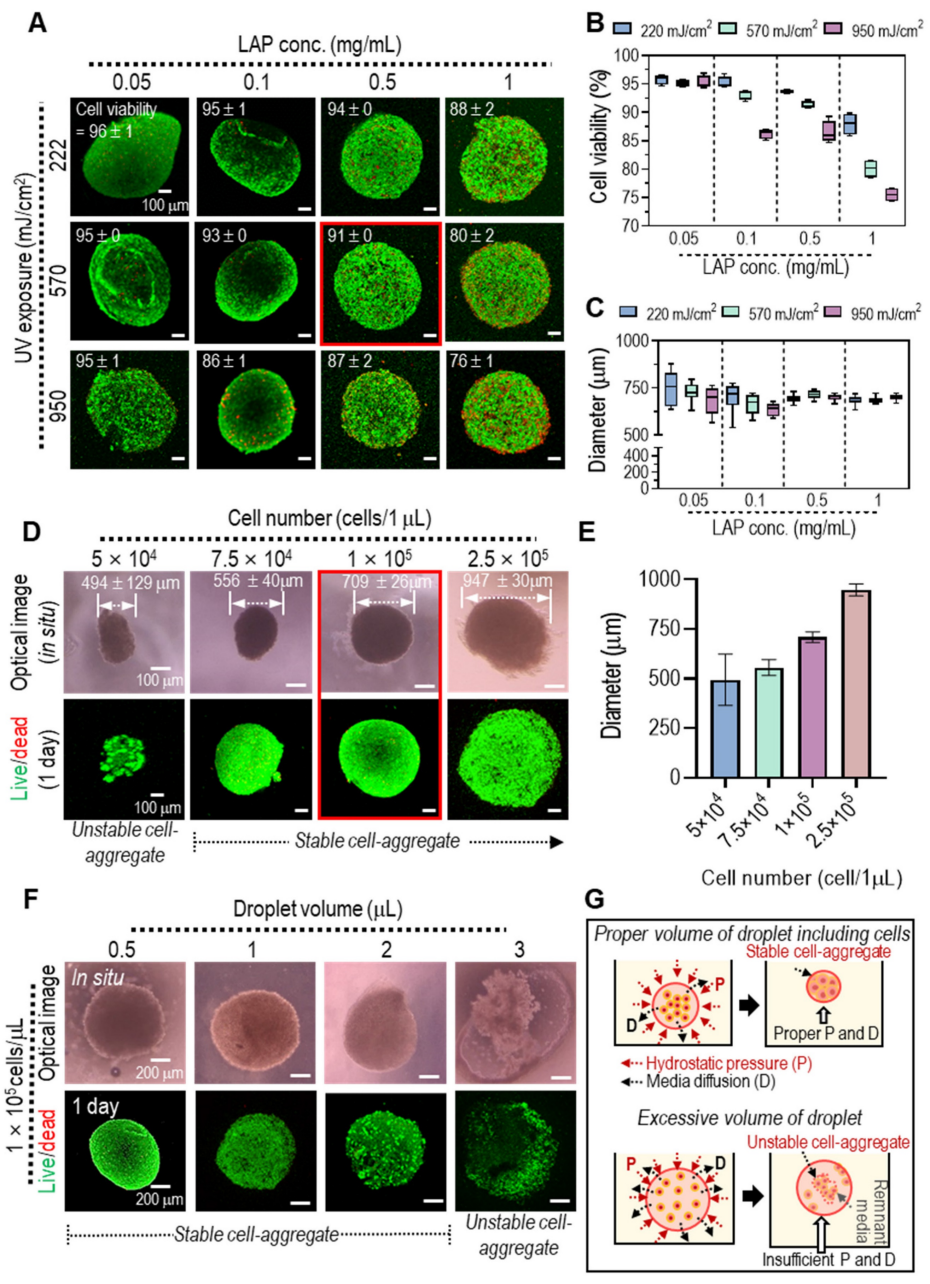
**(A)** Live (green) and dead (red) images of the cell aggregates using the Col-Ma-coated cells for various UV conditions (LAP and UV intensity) and **(B)** the cell viability and **(C)** diameter of the cell aggregates. **(D)** The optical and live/dead images of cell aggregates fabricated using various cell numbers in a 1-µL droplet and their average diameter. **(E)** The optical and **(F)** live/dead images of cell aggregates fabricated using various droplet volumes with a fixed cell number (1 × 10^5^). **(G)** Schematics to show the effect of droplet volume on the structural stability of the cell aggregate formation.

Generally, UV radiation can induce DNA damage in cells, potentially impacting cell viability 32, 33. To identify UV radiation parameters for achieving structurally stable and biologically safe Col-Ma-coated hASC aggregates, we explored various photoinitiator concentrations (0.05 to 1 mg/mL) and UV powers (222 to 950 mJ/cm^2^) on the Col-Ma-coated cells. **Figure 3A** shows the effect of various LAP concentration and UV power on live (green)/dead (red) and physically stable spherical shape formation, as determined by measuring the diameters of several two-dimensional cell aggregate images. With increasing LAP concentration and UV power, stable formation (low standard deviation) of cell aggregates was obtained (**Figure 3B**), whereas cell viability gradually decreased (**Figure 3C**). Based on the test showing the range of safe cell viability and physical spherical shape formation, we selected the appropriate crosslinking conditions (LAP concentration: 0.5 mg/mL and UV intensity: 570 mJ/cm^2^).

After selecting the UV crosslinking conditions to fabricate cell aggregates, we assessed the effect of cell number and cell droplet volume on cell viability and stable cell aggregate formation. When the cell number was increased from 5 × 10^4^ to 2.5 × 10^5^ in a fixed droplet volume of 1 µL, the average size of the cell aggregates gradually increased, and the cell viability (at 1 d) of all cell aggregates was safe (**Figure. 3D-E**). However, for a relatively low cell number (5 × 10^4^ in 1 µL droplet), the formation of cell aggregates was unstable. In addition, we assessed the effect of the cell droplet volume (0.5, 1, 2, and 3 µL) with a fixed cell number (1 × 10^5^) per droplet on cell viability and cell aggregate formation. As shown in the optical and live/dead images in **Figure 3F**, although all cell aggregates have high cell viability (over 90%), the relatively high droplet volume (3 µL) can cause the unstable formation of pseudo-spheroids owing to the poor contact between the coated cells, which can induce poor crosslinking of the Col-Ma-coated cells within the droplet, as shown in the schematic image in **Figure 3G**. Based on these results, we selected the appropriate cell density (1 × 10^5^/µL) for the droplet volume to fabricate stable cell aggregates. Additionally, we employed an identical fabrication process to generate pseudo-spheroids using diverse cell types, C2C12, Hs-27, Caco-2, and HepG2, shown in supporting information (**Figure S2**). In the optical and fluorescence images, the pseudo-spheroids composed of cells coated with a collagen matrix (Col-Ma) were successfully generated, exhibiting consistent formation regardless of the specific cell types employed.

### Comparison between the spheroids fabricated with the hanging drop method and the cell aggregates consisting of Col-Ma-coated cells

**Figure 4:**
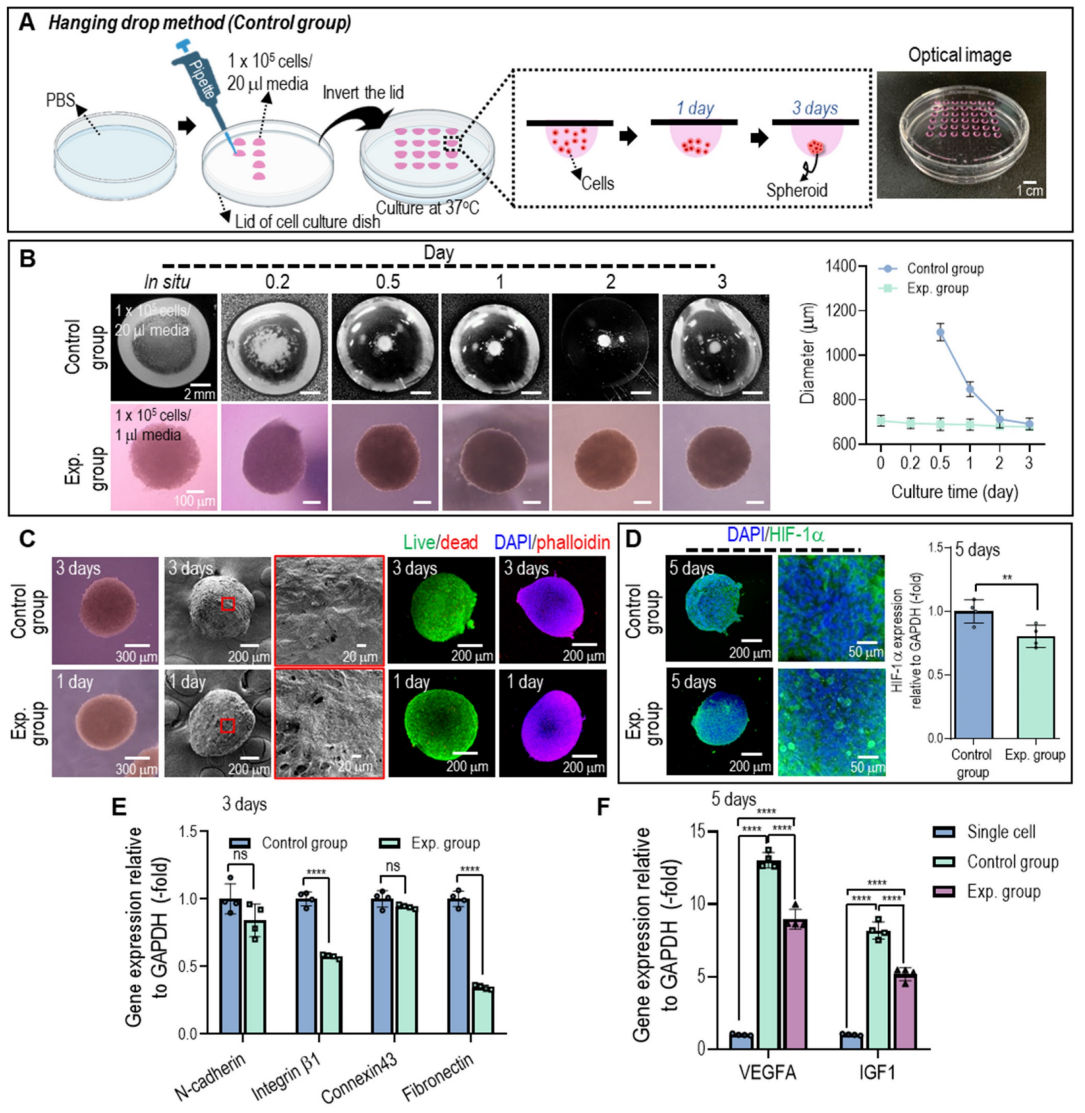
**(A)** Schematics and optical image to show the conventional hanging droplet method used to fabricate cell spheroids. **(B)** Comparison of the spherical formation for the cell spheroid (control) fabricated using the hanging drop and cell aggregate (experimental) using Col-Ma-coated cells and their diameter change vs. culturing period. **(C)** Optical, SEM, live/dead, and DAPI (blue)/phalloidin (red) images of the control and experimental groups on day 3 of the cell culture. **(D)** Fluorescence image of DAPI (blue)/HIF-1α (green) and its gene expression level for the control and experimental groups on day 5 of the cell culture. **(E)** The gene expression of control vs. experimental group on 3 days and **(F)** the comparison of VEGF and IGF gene for single cell, control, and experimental group on 5 days of the cell culture. (ns = no significance, ^**^ *p <* 0.005, and ^****^ *p <* 0.0001).

The hASC aggregates (experimental group) fabricated using the previously fixed processing conditions using Col-Ma-coated hASCs were compared with normal hASCs spheroids (control group) fabricated using the traditional hanging drop method to evaluate biological activities and spherical shape formation. The detailed fabrication conditions for the hanging drop method are shown in **Figure 4A**.

The average diameters and surface morphological shapes of the cell spheroids and aggregates were measured using light microscopy, SEM, and fluorescence imaging (live/dead and DAPI (blue)/phalloidin (green) images) for various cell culture periods, as shown in **Figures 4B** and** C**. As shown in the optical images in **Figure 4B**, the hASCs were scattered at the bottom of the droplet in the hanging drop method, and the cells aggregated into irregularly shaped spheroids for 2-3 days, whereas the cell aggregates fabricated using the method proposed in this study showed continuous stable formation after *in situ* fabrication. In the diameter measurement, three days after culturing, similar sizes (~700 ± 30 µm) were observed in both cell constructs. However, the optical images showed that the cell aggregate method provided a much more stable spherical shape, indicating that the size difference between *in situ* fabrication and three-day cell-culturing was nonsignificant. Furthermore, cell viability and cytoskeleton formation were similar, but cell aggregates (experimental, even after 1 d) exhibited much smoother surfaces than cell spheroids (control).

Cell spheroids larger than several hundred micrometers can induce hypoxic conditions in their central region owing to the limited diffusion of oxygen, leading to cell death in the core of the spheroid 31, 34. This hypoxic environment can activate the expression of HIF-1α, a transcription factor that plays a crucial role in cellular responses to low oxygen conditions. Therefore, evaluating the expression and activity of HIF-1α can provide insights into the cellular response to hypoxia in cell spheroids. As shown in **Figure 4D**, on day 5, both spherical cell structures (control and experimental) were stained with HIF-1α, and its gene expression was evaluated. HIF-1α gene expression in the cell aggregates fabricated using the proposed method was significantly lower than in the control. We assumed that the reason for the lower HIF-1α in the cell aggregates fabricated using the Col-Ma-coated cells could be that the thin Col-Ma layer coated on the cells could be a porous path, which could induce more efficient diffusion of nutrients and oxygen transport within the aggregated cells, lowering the hypoxia of the Col-Ma-coated cell aggregate.

In addition, to observe the genes related to cell-to-cell interactions in the 3D cell structures, cell adhesion molecules (N-cadherins and integrin β-1), connexin 43, which is involved in cell-to-cell communication, and an extracellular matrix protein (fibronectin) were assessed on day 3 (**Figure 4E**). The results showed that gene expression of the cell aggregates (experimental) was slightly lower than that of the conventionally fabricated cell spheroids (control), which may be due to the interference of the coated Col-Ma layer.

To compare the efficacy of cell spheroids and cell aggregates with single cells in terms of angiogenesis and growth factor signaling, we conducted gene expression measurements of VEGF and IGF on 5 days (**Figure 4F**). VEGF is primarily involved in angiogenesis, the process of forming new blood vessels that support muscle regeneration, and IGF has the potential to enhance the recruitment and differentiation of myoblasts, contributing to muscle tissue repair and regeneration. Although there was significant difference observed between the spheroids and cell aggregates, which is consistent with the findings related to genes associated with cell-cell interaction, the cell aggregates exhibited significantly higher expression levels of VEGF and IGF genes compared to single cells on 5 days. Furthermore, the expression of stemness-related genes (OCT4 and SOX2) in hASCs cultured in different environments suggests a significant upregulation in cell spheroids and aggregates compared to single cells **(Figure S3)**. These data imply that culturing hASCs in cell spheroids or aggregates has led to the promotion of pluripotent markers. Similarly, previous studies have indicated that the pluripotency of hASCs is upregulated when cultured in cellular spheroid form 35, 36.

The increased expression of VEGF and IGF in the aggregated cells suggests that these cell aggregates have the potential to promote muscle tissue regeneration. However, it is important to note that gene expression analysis alone does not provide a comprehensive understanding of the regenerative potential of cell-aggregates. Further investigations, including *in vivo* studies, are necessary to evaluate their effectiveness in muscle tissue regeneration and validate their therapeutic potential. Considering this, we applied the cell aggregates in a mouse model of volumetric muscle loss to gain understandings into its regenerative capabilities.

### Fabrication of cylindrical cell constructs using a normal bioprinting process and cell aggregate process

**Figure 5:**
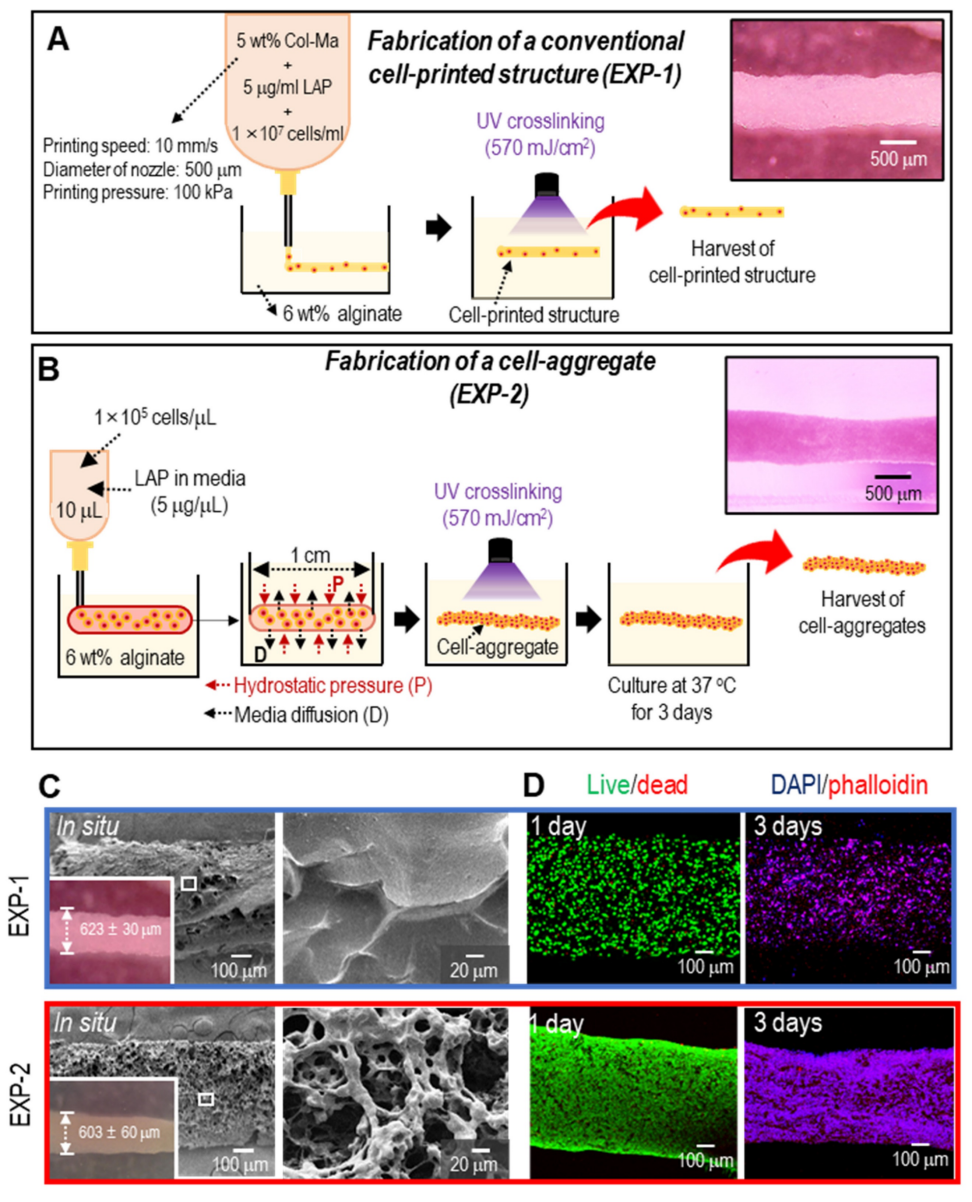
Schematics of fabricating **(A)** a cell-laden strut (EXP-1) using a submerged bioprinting process and **(B)** a cylindrical cell aggregate (EXP-2) obtained using Col-Ma-coated cells and cell-aggregating process. **(C)** Optical and SEM images of fabricated EXP-1 and EXP-2. **(D)** Live/dead on day 1 and DAPI/phalloidin images on day 3 of the fabricated cell constructs.

The findings of this study suggest that Col-Ma-coated hASC aggregates can be an efficient approach for tissue regeneration compared with bioprinted hASC-laden struts. To expand the application of the proposed cell aggregates to muscle tissue regeneration, we fabricated normally bioprinted cell-laden struts (EXP-1), which were printed in a bath filled with a 6 wt% alginate solution, as shown schematically in **Figure 5A**. As shown in **Figure S4A** and** B**, a higher concentration of Col-Ma corresponded to a greater diameter of EXP-1 struts. As a comparable structure, we fabricated cylindrical cell aggregates (EXP-2; diameter = 603 ± 60 µm and length = 10 ± 1 mm) using Col-Ma-coated cells (cell number = 1 × 10^5^/µL and droplet volume = 10 µL) and hASCs (**Figure 5B**). The detailed fabrication conditions for both methods are shown in **Figures 5A** and **B**. **Figures 5C** and **D** show the optical/SEM/live-dead and DAPI/phalloidin staining of the fabricated structures (EXP-1: bioprinted hASC-laden structure and EXP-2: Col-Ma-coated hASC aggregate). Both cylindrical structures were geometrically similar; however, the surface roughness differed because of the Col-Ma layer coated on the cells in EXP-2. In addition, as revealed by the live/dead images on day 1, both cell constructs exhibited high cell viability (>90%), indicating that both processes are safe (**Figure 5D**). However, cell morphology on day 3 was different between the two groups (**Figure 5D**). The EXP-1 group, with individually dispersed hASCs, showed relatively poor cell-cell interactions, whereas the EXP-2 group displayed much more active cytoskeleton development, indicating a high degree of cell-cell interactions.

### *In vivo* experimental

**Figure 6:**
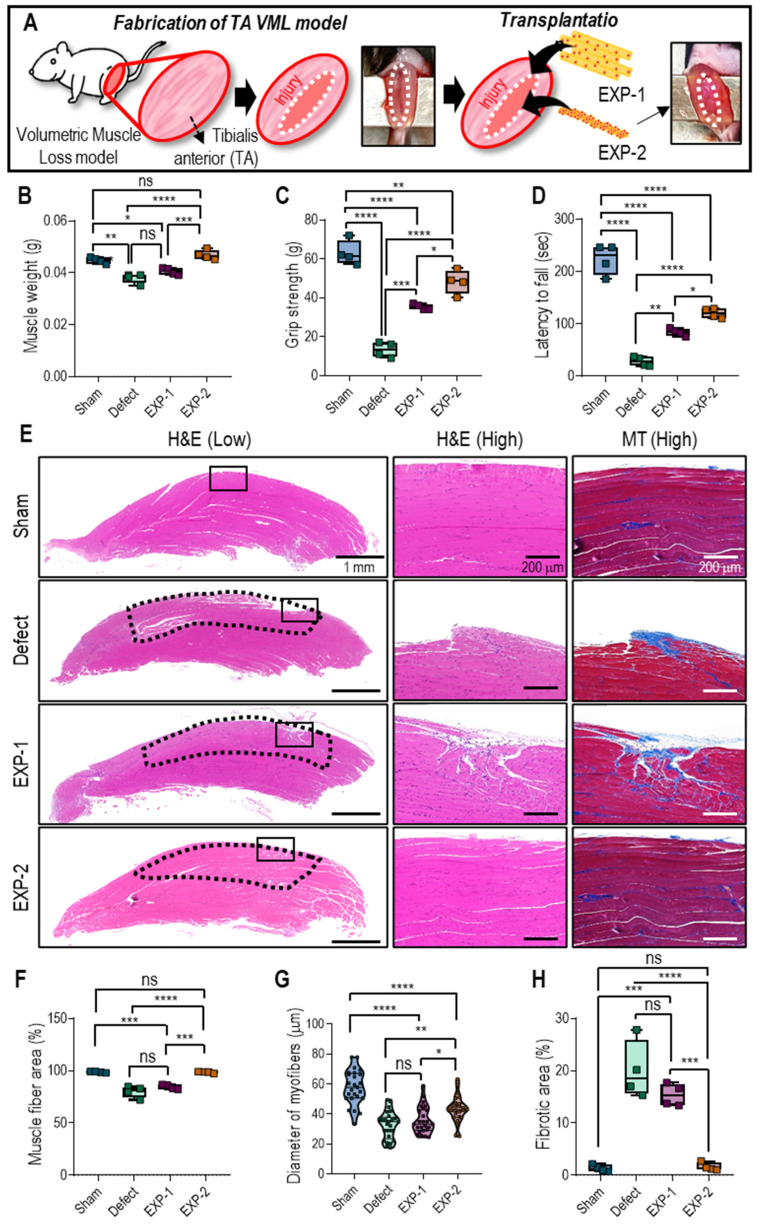
** (A)** Schematic of implantation of hASC-laden cell construct on VML defect on TA. **(B)** Muscle weight, **(C)** hindlimb grip strength, and **(D)** latency to fall at four-week post-implantation. **(E)** H&E and MTS staining of transplanted sites (black dotted line indicates the defect region). Calculated **(F)** muscle fiber area (%), **(G)** myofiber diameter, and **(H)** fibrotic area (%). (*n* = 4, ns = no significance, ^*^ *p <* 0.05, ^**^ *p <* 0.005, ^***^ *p <* 0.001, and ^***^ *p <* 0.0001).

In this study, a VML model was generated in mice by eliminating the extensor digitorum longus/extensor hallucis longus and inducing a 40% reduction in the TA muscle, leading to an irreparable volumetric deficiency. After implanting the muscle constructs, which were fabricated using the cylindrical cell structures (EXP-1 and EXP-2) in the VML deficit mice, they were assessed for four weeks. For cell constructs of the same size, the total number of cells in the bioprinted struts differed from that in cell aggregates. Therefore, the total number of cells used in the experiment remained unchanged after adjusting the number of bioprinted struts (**Figure 6A**).

After four weeks of implantation of muscle constructs (EXP-1 and EXP-2), muscle regeneration was assessed based on muscle weight (**Figure 6B**), functional tests (**Figures 6C-D**), and histological staining (**Figure 6E**). The muscle weight of the EXP-2 group (Col-Ma-coated hASC aggregate) was significantly higher than that of the nontreated (defect) and EXP-1 (hASC-laden Col-Ma strut) groups and comparable to that of the sham group that did not have TA loss induced, as shown in **Figure 6B**. Muscle function was evaluated using hindlimb grip strength and latency to fall tests. The grip strengths were as follows: 63 ± 6 g (sham), 13 ± 4 g (defect), 35 ± 2 g (EXP-1), and 48 ± 6 g (EXP-2). The latency to fall from the groups was: 223 ± 29 s (sham), 28 ± 9 s (defect), 84 ± 7 s (EXP-1), and 120 ± 9 s (EXP-2). The outcomes indicated a significant improvement in muscle function in the EXP-2 group compared with the other groups, as demonstrated by the increased latency to fall and grip strength. The muscle function of the EXP-2 group was relatively close to that of the sham group.

To assess the muscle regenerative efficacy of the constructs, cross-sections from each group were stained with H&E and MT to quantify muscle fiber formation and collagen deposition (**Figure 6E**). The EXP-2 construct resulted in efficient volumetric muscle regeneration with significantly more mature regenerating muscle fibers (muscle fiber area, **Figure 6F**) than the nontreated group. Muscle fiber diameters were as follows: 58 ± 11 µm (sham), 32 ± 9 µm (defect), 36 ± 9 µm (EXP-1), and 43 ± 9 µm (EXP-2) (**Figure 6G**). Excess collagen deposition, measured as fibrotic areas, was significantly higher in the nontreated and EXP-1 groups (20 ± 6% and 15 ± 2%, respectively) than in the EXP-2 group (2 ± 1%; **Figure 6H**).

**Figure 7:**
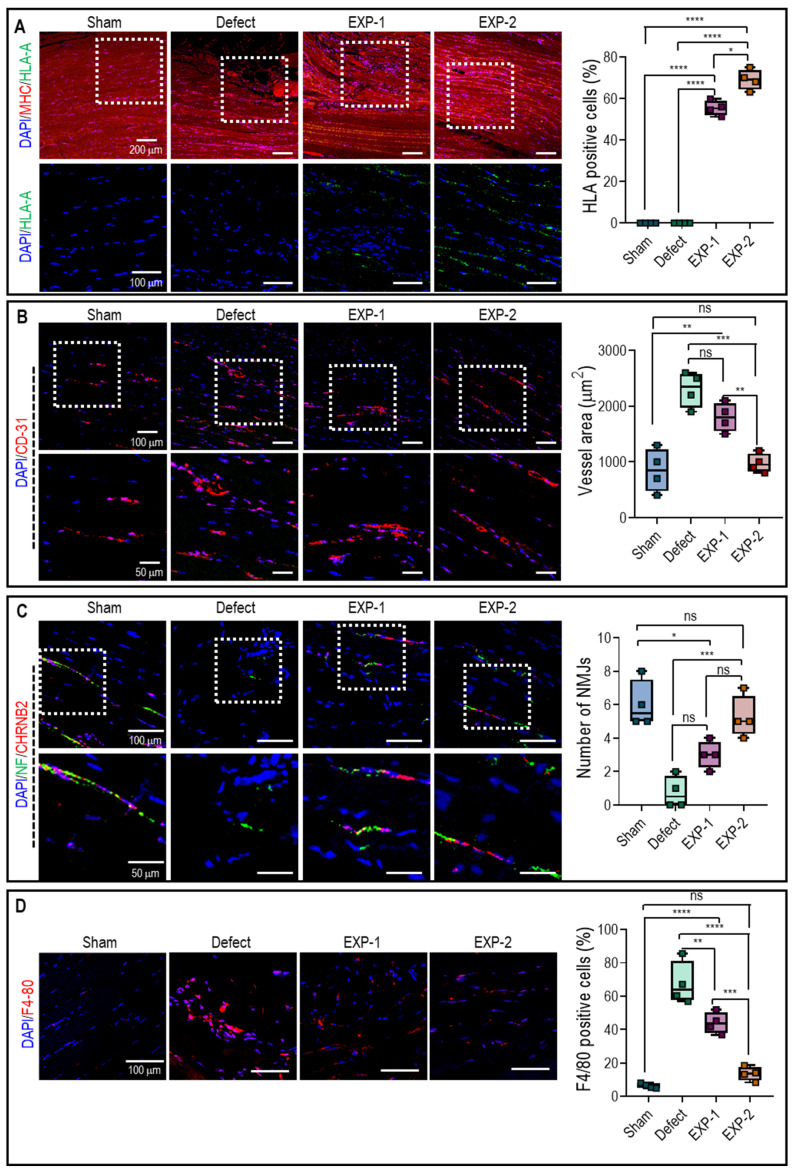
Immunochemical staining of **(A)** DAPI (blue)/MHC (red)/HLA-A (green) and percentage of cells with positive HLA-A, **(B)** DAPI/CD31 (red) and vessel area, **(C)** DAPI/CHRNB2 (red) and number of NMJs, and **(D)** DAPI/F4-80 (red) and percentage of cells with positive F4-80. (*n* = 4, ns = no significance, ^*^ *p <* 0.05, ^**^ *p <* 0.005, ^***^ *p <* 0.001, and ^****^ *p <* 0.0001).

To investigate the contribution of implanted hASCs to muscle regeneration within bioengineered tissue constructs, sectioned TA muscles were stained with DAPI/MHC/HLA and analyzed (**Figure 7A**). As expected, HLA that has human-specific reactivity was expressed in both implanted groups, but not in sham and defect groups. The positive expression of HLA and MHC suggested that hASCs within the muscle constructs could differentiate and form myofibers. Moreover, the number of HLA-positive cells was significantly higher in the EXP-2 group than in the EXP-1 group, indicating that larger quantities of hASCs have integrated with native muscle tissues.

Rapid vascular ingrowth is crucial to avoid necrosis of transplanted cells owing to limited oxygen and nutrient supply 37, 38. In this study, host vascular ingrowth into the implanted constructs was evaluated by assessing CD-31 expression (**Figure 7B**). Immunofluorescence images and vessel number analysis showed that the vessel number in the EXP-2 group was similar to that in the sham group, likely due to the mature myofibers, whereas the defect and EXP-1 showed a larger vessel area due to the continued requirement of vascularization in muscle restoration 38.

Additionally, the development of NMJ in the implanted constructs was evaluated for functional restoration using double immunofluorescence for NF and *CHRNB2* (**Figure 7C**). The results showed that more NMJ were found in the EXP-1 and EXP-2 groups than in the defect group after implantation, but non-significant NMJ was observed between EXP-1 and EXP-2 group. Generally, the process of NMJ formation is complex and requires the coordination of various molecular and cellular events, so that the duration of four weeks may not have been sufficient for significant NMJ development to occur.

F4-80 staining, a widely used marker for macrophages, enables the visualization and quantification of macrophages within the tissue. Generally, a higher expression of F4-80 indicates increased infiltration and accumulation of macrophages. As shown in **Figure 7D**, F4-80 staining was significantly higher in the defect and EXP-1 groups than in the EXP-2 and sham groups. These results suggested that the defective and EXP-1 groups triggered a robust immune response, leading to macrophage recruitment and activation. However, in the EXP-2 group, muscle regeneration appeared to have reached an advanced stage similar to that observed in the sham group. The results reveal that the expressions of both MHCII and CD206 were significantly more pronounced in mice that received the defect and EXP-1 treatment compared to EXP-2 **(Figure S5)**. In addition, the similarity in expressions observed in mice receiving the EXP-2 bioconstruct after the VML defect, resembling those of the SHAM group, provides additional evidence for the advanced stages of muscle regeneration.

Based on the *in vivo* findings, it can be inferred that Col-Ma-coated cell aggregates exhibited efficient vascularization, resulting in blood vessels comparable to those observed in the sham group, and relatively low immune response. These alterations have the potential to accelerate muscle function restoration.

## Conclusion

Herein, we developed a new method for fabricating pseudo-cell spheroids (cell aggregates) by coating cells with Col-Ma and utilizing a submerging printing approach. Our approach demonstrates meaningful efficiency in fabricating cell aggregates, as it promotes the formation of physically stable structures and biologically active pseudo-spheroids. This is achieved using a porous Col-Ma layer, which can induce more efficient metabolic activities of cell aggregates compared to conventionally fabricated cell spheroids. However, it is important to carefully select appropriate fabrication components or conditions, such as Col-Ma concentration and photo-crosslinking conditions, to successfully fabricate Col-Ma-assisted cell aggregates. We utilized the proposed cylindrical hASC aggregates in a volumetric muscle defect mouse model to demonstrate the feasibility of Col-Ma-assisted pseudo-spheroids for tissue engineering applications. *In vitro* and *in vivo* studies showed meaningful cellular activities and muscle regeneration of the proposed cell aggregates compared with conventionally bioprinted struts with a similar cell density. Our study suggests that the newly developed cell aggregation method has significant potential for various tissue engineering applications.

## Supplementary Material

Supplementary figures and table.
