# Supplementary material for: Macroscale pseudo-spheroids fabricated using methacrylated collagen-coated cells
Source: Theranostics. 2024 Jan 1;14(3):924–39. doi: 10.7150/thno.92193 (PMC10797297; doi:10.7150/thno.92193)
Supplement: Supplementary file 1 — Supplementary figures and table. [file thnov14p0924s1.pdf]

Supporting information

Macroscale pseudo-spheroids fabricated using methacrylated collagen-coated cells

SooJung Chae[1], Hyeongjin Lee[2], Dongryeol Ryu[3], GeunHyung Kim[1,4,5*]

[1]Department of Precision Medicine, Sungkyunkwan University School of Medicine (SKKU-SOM) Suwon 16419, Republic of Korea

[2]Department of Biotechnology and Bioinformatics, Korea University, Sejong, Republic of Korea

[3]Biomedical Science and Engineering, Gwangju Institute of Science and Technology, Gwangju, Republic of Korea.

[4]Institute of Quantum Biophysics, Department of Biophysics, Sungkyunkwan University, Suwon, Gyeonggi-do 16419, Republic of Korea

[5]Biomedical Institute for Convergence at SKKU (BICS), Sungkyunkwan University, Suwon 16419, Republic of Korea

[*]Corresponding author

Prof. GeunHyung Kim, Tel: +82-31-290-7828; E-mail: gkimbme@skku.edu

Table S1. The weight of the total and removed 40% of tibialis anterior (TA) muscle in C57BL/6 mouse.

Number of mice	TA muscle weight (g)	Removed 40% of the TA muscle weight (g)
1	0.0441	0.0176
2	0.0449	0.0180
3	0.0411	0.0164
4	0.0458	0.0184
5	0.0439	0.0176
6	0.0452	0.0181
7	0.0501	0.0200
8	0.0442	0.0177
9	0.0442	0.0177
10	0.0398	0.0159
11	0.0407	0.0163
12	0.0445	0.0178
13	0.0414	0.0166
14	0.0426	0.0170
15	0.0392	0.0157
16	0.0431	0.0172
Mean ± SD	0.0434 ± 0.0027	0.0174 ± 0.0011

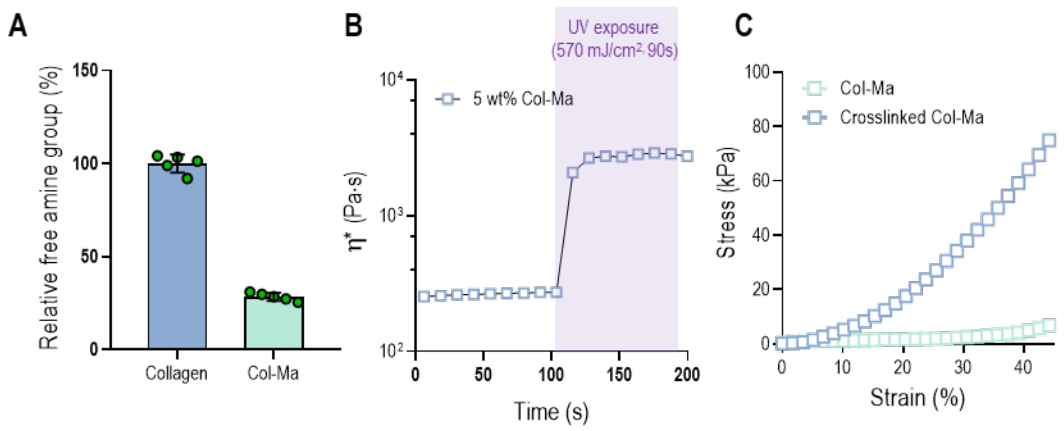

Figure S1. (A) Relative free amine group evaluated using ninhydrin assay. **(B)** Complex viscosity (η*) and **(C)** stress-strain curve of 5 wt% Col-Ma and after UV photocrosslinking.

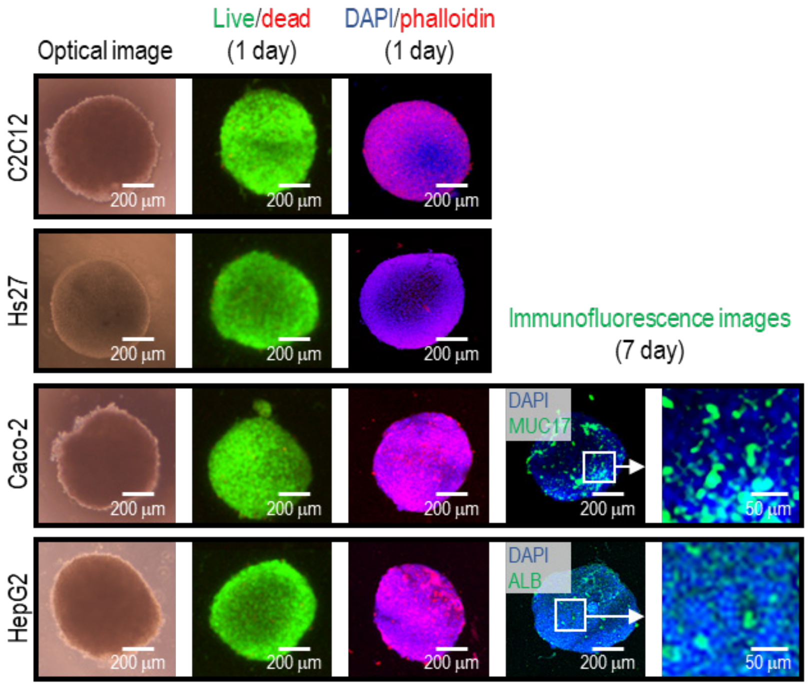

Figure S2. Pseudo-spheroids fabricated with Col-Ma-coated C2C12, Hs-27, Caco-2, and HepG2, which were obtained with the same fabrication condition of hASCs-aggregates.

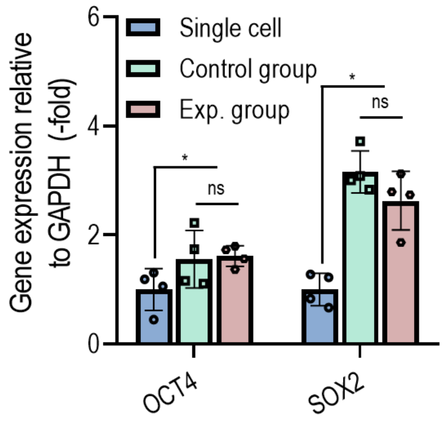

Figure S3. Relative gene expressions of stemness-related genes (OCT4 and SOX2) for a single cell, control, and experimental group after 1 day of cell culture. (n = 4, ns = no significance, $^{*}p < 0.05$, $^{**}p < 0.005$, $^{***}p < 0.001$, and $^{****}p < 0.0001$).

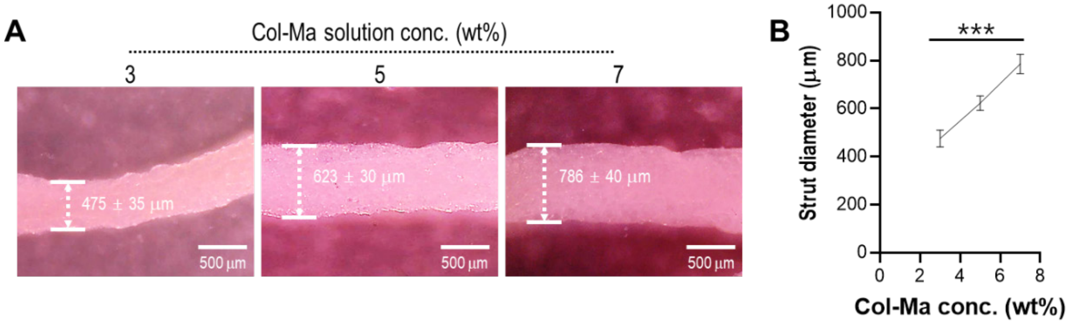

Figure S4. (A) Optical image and **(B)** measured strut diameter of fabricated cell-laden struts (EXP-1) using various concentrations of Col-Ma solutions. (n = 50, ns = no significance, $^{*}p < 0.05$, $^{**}p < 0.005$, $^{***}p < 0.001$, and $^{****}p < 0.0001$).

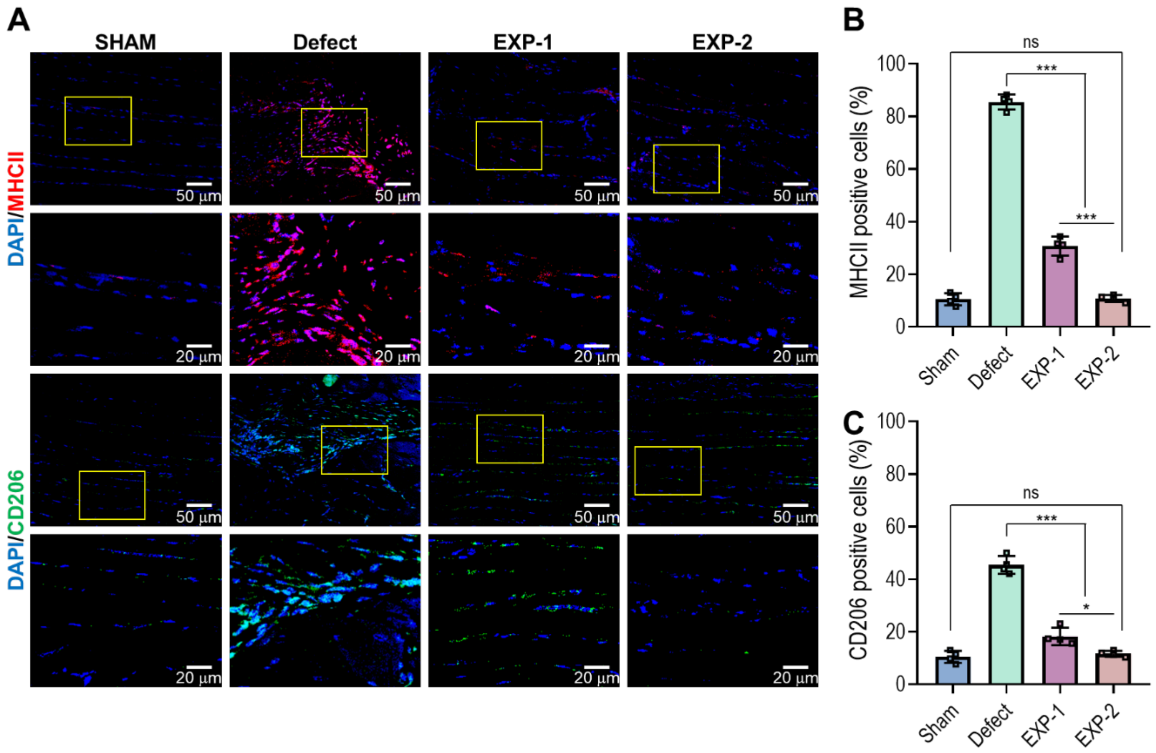

Figure S5. Immunochemical staining of **(A)** DAPI (blue)/MHCII (red) and DAPI (blue)/CD206 (green). Percentage of cells with positive **(B)** MHCII and **(C)** CD206. (n = 4, ns = no significance, $^{*}p < 0.05$, $^{**}p < 0.005$, $^{***}p < 0.001$, and $^{****}p < 0.0001$).
